# Patients' perspectives on how idiopathic pulmonary fibrosis affects the quality of their lives

**DOI:** 10.1186/1477-7525-3-61

**Published:** 2005-10-07

**Authors:** Jeffrey J Swigris, Anita L Stewart, Michael K Gould, Sandra R Wilson

**Affiliations:** 1 Division of Pulmonary Medicine, Interstitial Lung Disease Program, National Jewish Medical and Research Center, Denver, CO, USA; 2 University of California San Francisco, San Francisco, CA, USA; 3 VA Palo Alto Health Care System, Palo Alto, CA, USA; 4 Palo Alto Medical Foundation Research Institute, Palo Alto Medical Foundation, Palo Alto, CA, USA; 5 Division of Pulmonary and Critical Care Medicine, Stanford University, Stanford, CA, USA

**Keywords:** Pulmonary fibrosis, interstitial lung disease, quality of life, Health-related quality of life, qualitative.

## Abstract

**Background:**

Idiopathic pulmonary fibrosis (IPF) is a debilitating lung disease with a survival of only three to five years from the time of diagnosis. Due to a paucity of studies, large gaps remain in our understanding of how IPF affects the quality of patients' lives. In only one other study did investigators ask patients directly for their perspectives on this topic. Further, currently there is no disease-specific instrument to measure health-related quality of life (HRQL) in patients with IPF. A carefully constructed measurement instrument, sensitive to underlying change, is needed for use in clinical trials and longitudinal studies of patients with IPF. Before developing such an instrument, researchers must improve their understanding of the relevant effects of IPF on patients' lives. On a broader scale, to provide the best care for people with IPF, clinicians must appreciate – from patients' perspectives – how this disease affects various aspects of their lives.

**Methods:**

We used focus groups and individual in-depth interviews with 20 IPF patients to collect their perspectives on how IPF affects their lives (with a focus on the quality of their lives). We then analyzed these perspectives and organized them into a conceptual framework for describing HRQL in patients with IPF. Next, we examined how well certain existing measurement instruments – which have been administered to IPF patients in prior studies – covered the domains and topics our patients identified.

**Results:**

In our framework, we identified 12 primary domains: symptoms, IPF therapy, sleep, exhaustion, forethought, employment and finances, dependence, family, sexual relations, social participation, mental and spiritual well-being, mortality. Each domain is composed of several topics, which describe how IPF affects patients' lives. When we compared the content of our conceptual framework with the existing instruments, we found the coverage of the existing instruments to be inadequate for several reasons, including they may tap general areas of QOL or HRQL but not some areas that appear to be most directly affected by IPF, and they include items that are relevant to symptoms and effects of other respiratory diseases but not IPF.

**Conclusion:**

Collecting patients' perspectives and developing an organized inventory of the relevant effects of IPF on patients' lives provides valuable information for improving our understanding of the impact of this disease on patients and their loved ones. We believe our findings will help alert clinicians and researchers to IPF patients' experiences and concerns. Based on the comparison or our conceptual framework with the content of four existing instruments, it would appear that developing an IPF-specific measurement instrument is justified. Our conceptual framework for describing health-related quality of life in patients with IPF lays a solid foundation for constructing such an instrument.

## Background

Idiopathic pulmonary fibrosis (IPF) is the most common of the idiopathic interstitial pneumonias. It is thought to affect about 30 persons per 100,000 people in the general population and perhaps as many as 175 per 100,000 people 75 years and older [1,2]. Breathlessness and irritating dry cough, often refractory to anti-tussive therapy, are classic symptoms. The disease course of IPF is somewhat variable, but patients commonly suffer progressive decline in lung function that culminates in respiratory failure and death. Median survival ranges from three to five years from the time of diagnosis [3-5]. Conventional IPF pharmacotherapy, which includes corticosteroids (i.e., prednisone) in combination with an immunosuppressive agent (e.g., azathioprine or cyclophosphamide), is largely ineffective, fraught with adverse effects, and often requires frequent laboratory monitoring [6].

The definition for quality of life (QOL) that we use in this manuscript refers to an individual's "holistic" evaluation of satisfaction with his own life [7]. Health-related quality of life (HRQL) incorporates the subjectively perceived impact of one's health – including aspects of well-being (or lack thereof) in the physical, mental, emotional, social, and spiritual facets of life – on life domains of perceived importance. Currently, there is no instrument that is claimed to be appropriate for evaluating HRQL specifically in patients with IPF. A handful of studies [8-13], using existing generic or non-IPF respiratory-specific measurement instruments, have assessed QOL or HRQL in patients with IPF, but none of these instruments have been shown to be sensitive to disease progression or to treatment effects (although detecting the latter presupposes the existence of effective treatment, which unfortunately is not currently available in the case of IPF). The generic measurement instruments that have been used with IPF patients include the World Health Organization 100-Item Instrument (WHOQOL-100) [14], the Quality of Well-Being Scale (QWB) [15], and the Medical Outcomes Study Short-Form 36-Item Instrument (SF-36) [16] – none of which were specifically designed for patients with devastating illnesses like IPF. Further, in a cross-sectional study of 50 patients with various interstitial lung diseases, including 33 with IPF, Chang and her colleagues [8] found that the QWB's content and scaling made it incapable of distinguishing patients with varying degrees of IPF severity.

The respiratory-specific measures administered to IPF patients in prior studies included the Chronic Respiratory Questionnaire (CRQ) [17] and St. George's Respiratory Questionnaire (SGRQ) [18] – both of which were designed specifically for patients with obstructive lung diseases (e.g., asthma, chronic bronchitis, and emphysema) [8-13]. Idiopathic pulmonary fibrosis lies within a completely different spectrum of diseases from these; its physiologic hallmark is ventilatory restriction, not obstruction. The symptoms of IPF are different from the symptoms of obstructive diseases, which cause wheezing, productive cough, and attacks of disease activity: IPF does not cause wheezing, its cough is typically not productive, and IPF symptoms are not episodic. In the same study mentioned above, Chang and her co-investigators [8] noted that the original form of the CRQ underestimated the negative impact of breathlessness on their IPF patients' quality of life because that instrument allowed patients to rate their dyspnea during self-identified activities. Patients whose activity level had become increasingly restricted were rating their breathlessness while performing less taxing activities; hence, the true impact of breathlessness was not reflected in their scores.

In only one study, which enrolled ten patients, did investigators directly ask patients for their perspectives on how IPF impacted their lives [11]. However, the ultimate aim of that study was to assess the relevance of two measurement instruments for patients with IPF. The investigators did not intend to inventory the myriad effects of IPF on the quality of patients' lives or to develop a conceptual framework for describing quality of life in patients with IPF. Such objectives require study in a more systematic manner and on a somewhat larger scale. Further, additional study is required to properly assess the adequacy of current instruments for evaluating patients with IPF, and, if the instruments are found to be inadequate, to provide a basis for developing a more appropriate instrument to serve this purpose.

We conducted the present study to achieve four objectives: 1) to gain further insight into the effects of IPF on patients' lives – from their own perspectives, 2) to organize those effects into a structured conceptual framework, 3) to examine whether four existing instruments adequately cover the elements of this framework, and 4) to examine the extent to which the four existing instruments include items that may be irrelevant to patients with IPF – or that may not serve well the purpose of evaluating the impact of IPF progression and the effects of disease treatment on IPF patients' quality of life.

## Methods

We recruited from the general pulmonary and Interstitial Lung Disease Clinics at Stanford University patients with IPF and invited them to participate in focus group meetings or individual in-depth interviews to discuss how IPF affects their lives. Such qualitative data are often collected using multiple methods, including focus groups, key informant interviews, expert opinion, and clinical observation. To improve data capture, we decided to collect patients' perspectives using two formats – focus groups and interviews. An interview accommodates those patients who are unwilling or unable to participate in a focus group. For example, in some cases, patients are willing to freely share information in an individual interview that they might not share in a group setting, while a focus group allows participants to react and respond to the other members and to build on ideas raised during the session. We selected a heterogeneous sample to capture the views of patients in each stage of disease and with varying times since diagnosis. To identify the number of patients to enroll in the study, we used the process of "sampling to redundancy"; in other words, we conducted interviews and focus groups with different patients until no new themes or effects emerged [19]. We ended up with 20 patients in our sample. For each participant, the diagnosis of IPF was confirmed using currently accepted criteria [1]. Patients with other causes of lung fibrosis were not eligible.

To ensure that we addressed all major categories (dimensions) of general quality of life, two investigators (JS and SW) developed a brief set of questions, based loosely on Flanagan's Quality of Life Scale, which were used in the focus groups and interviews [20]. One (JS) or two (JS and SW) investigators took notes and moderated the focus groups and interviews, which were conducted between September 2003 and February 2004. Each of the three focus groups lasted approximately two hours. Each of the five individual interviews lasted approximately one hour. Groups and interviews were audio-taped and transcribed. The study protocol was approved by the Stanford University Institutional Review Board, and all the participants provided informed, written consent prior to enrollment.

## Analysis

In the first step of the analysis, we divided the transcripts into individual text units, defined as identifiable segments of continuous speech, ranging in size from phrases to entire paragraphs that identified some effect of IPF on an individual's life. Using NVivo qualitative analysis software (QSR International Pty. Ltd.), we formed sub-categories by clustering identical text units, or ones that addressed essentially the same concept. We then grouped similar sub-categories to form primary conceptual categories (domains of IPF-related quality of life). Thus, the domains include sub-categories that are all distinct from each other and that are comprehensive of all the unique effects mentioned by patients in the groups and interviews.

We then compared the topics and specific item content of the WHOQOL-100, SF-36, CRQ, and SGRQ (measurement instruments administered to IPF patients in previous studies) with the domains that we identified in our analysis. In this process, we examined how well these instruments reflected the identified effects of IPF on patients' lives and whether they contained items that were – based on our patients' perceptions – either not relevant or of questionable utility for assessing change due to disease progression or in response to therapy for IPF.

## Results

Demographic data and selected clinical characteristics of the participants are presented in Table 1. Our sample consisted of 13 men than 7 women. Most patients had their diagnoses confirmed by surgical lung biopsy, and all but one patient was taking at least one medication specifically for IPF.

**Table 1:** Demographic and clinical characteristics of IPF patients

**Characteristic**	**Distribution**
**Gender**	
male	13
female	7
**Age**	67 yrs (44–82 yrs)*
**Years since diagnosis**	1.8 yrs (0.67–11 yrs)*
**Mode of diagnosis**	
Via surgical biopsy	14
Via clinical criteria	6
**Supplemental oxygen use**	
No use	6
Use with exertion and sleep	4
Continuous use	10
**Comorbid conditions**	
Cured prostate cancer	1
Stable coronary artery disease	1
Chronic obstructive pulmonary disease	1
Paroxysmal atrial fibrillation	1

*Data presented as median and (range).

Our analysis produced a conceptual framework consisting of 12 domains. The definitions of these domains are presented in Table 2, and a narrative summary of some of the qualitative data substantiating each domain is provided in this section.

**Table 2:** Definitions of domains in conceptual framework for describing HRQL in Patients with IPF

**Domain**	**Definition**
1. Symptoms	Amount, severity, and impact of cough and breathlessness; impact of symptoms on physical functioning
2. IPF Therapy	Feelings toward medications and impact of medications on physical and mental health; supplemental oxygen use and impact on quality of life
3. Sleep	Quality and quantity of sleep; impact of sleep disturbance
4. Exhaustion	Lack of energy; feeling exhausted; impact of energy/exhaustion on quality of life
5. Forethought	Need to plan and prepare for activities before undertaking them; others' appreciation for the amount of planning and preparation required; impact of need for forethought on quality of life
6. Employment and Finances	Effects on employment status and financial security
7. Dependence	Need to rely/depend on other people; need to ask for help; fear of being a burden
8 Family	Impact of disease on family and relationships with family members
9. Sexual Relations	Limitations on sexual activity and sexuality; impact of impaired sexual relations on quality of life
10. Social Participation and Leisure Activities	Impact on functioning in relationships, social interactions; social isolation; attention to use of time
11. Mental and Spiritual Well-being	Psychological effects including fear, worry, anxiety; problems concentrating/focusing; effects on spirituality/spiritual self
12. Mortality	Feelings about death and dying; thoughts on mortality; impact on quality of life

### Symptoms

Not surprisingly, symptoms (shortness of breath and cough) were mentioned as significant impairments to overall quality of life. Patients in the earlier stages of the disease were less breathless than those in the later stages. The latter patients noted that shortness of breath was extremely distressing, curtailed all physical activity, and made "even brushing my teeth an exertion". Participants performed physical activity of any kind less often and less intensely because of breathlessness. They noted having to "pause for at least five minutes just to catch my breath" while performing even simple tasks. They were breathless "carrying groceries...carrying anything", taking a shower, bending at the waist, and stooping. Cough was also very bothersome. It was frequently described as "dry and non-productive" or "hacking" and "occurring when I talk for long periods". Several participants mentioned having "a nagging desire to cough constantly" and "never feeling relieved after coughing".

### IPF Therapy

Because of the extremely low likelihood of a sustained beneficial response, and because of the high rates of bothersome side effects, most patients perceived conventional IPF therapies as being difficult to tolerate and, in many ways, "worse than the disease itself". Those patients who stopped taking certain conventional therapies were "glad to get rid of [those] medications". Nearly every participant voiced a willingness to be a "medical guinea pig" by taking novel experimental therapies for IPF. Patients who used supplemental oxygen felt "tied to the hoses" that supplied it. Having to fill their car trunks with oxygen tanks and experiencing various types of distress due to this visible indicator of their disability limited their willingness and ability to leave their homes, to participate in social activities, or travel.

### Sleep

Perceptions of how IPF impacted sleep ranged from no effect to nightly disturbance. Participants reported occasionally being entirely unable to fall asleep at night or being awoken in the middle of the night because of their cough.

### Exhaustion

Participants experienced low energy and feelings of exhaustion, which were distinct from the sensation of breathlessness. Exhaustion and "overwhelming fatigue" were very prominent and "as bothersome as breathlessness". Many noted a "consistent lack of energy", an ongoing "gradual decline in energy", and a need to "economize energy" during the day. Because of low energy, they "rest up, do part of the chore, and rest up again" to accomplish many of the things they want or need to do. Several patients mentioned feeling "completely wiped out" at the end of a normal day. Many mentioned that even their creative energy was low.

### Forethought

Participants described a need to "analyze every activity" before starting it and noted that IPF forced them to plan every activity throughout the day. They pored over excursions away from home: "Does this restaurant have a ramp [making pulling an oxygen tank easier]?", "How far away is the parking lot from the door?". They mapped out their routes through the grocery store to get needed items without unnecessary exertion. One woman described making "dry runs" – scouting out the driving distance, parking lots, and entrances to buildings – a day prior to her engagements.

### Employment and finances

In terms of their occupations or jobs, participants fell into three categories. They either: (1) had already retired prior to being diagnosed with IPF, (2) could not retire because their medical costs were so great, or (3) were disabled or lost [their] job/career because of IPF. Some of the patients who were still working felt the need to conceal their chronic illness from business colleagues, because the patients believed it made them "appear weak." One man summed up many of the patients' fears about financial insecurity by saying that "in terms of using up my finances, continuing living is a real concern of mine." In general, participants did not want to exhaust family savings on their medical care.

### Dependence

The near certainty of disease progression made participants sad and fearful, especially of "becoming more dependent on loved ones". Most said that "the least satisfying aspect of my life is not being as independent as I once was", and many noted that having IPF caused them a "loss of privacy" due to the need for assistance. Having someone else assist with bathing was incredibly worrisome for them. All of them (young and old) relied on someone or something just to "get by" (e.g., hand-rails in the shower, raised toilet seats). They were very frustrated by this and even more so by the likelihood that they will "become a physical or financial burden to family members".

### Family

Most participants mentioned an increased appreciation for the relationships they had with family members and the love and support that family members gave them throughout the course of their disease. Many said that the most satisfying part of their current lives was family. Some, however, mentioned that IPF strained their relationship with their spouse or significant loved ones. The limitations that IPF imposed caused many couples to completely change and rearrange their lifestyles; this was extremely frustrating, saddening, and stress-inducing. Many participants also found positive aspects of having IPF, including that it gave them the motivation and opportunity to spend more quality time with their family members.

### Sexual relations

Patients experienced decreased libido and a substantial curtailment of sexual activity, mostly due to diminished physical stamina. Several mentioned that their sexual partners were hesitant to engage in sexual activity or refrained from sexual activity altogether because of concerns about the patient over-exerting him- or herself. Many mentioned feeling less sexually attractive or desirable because of having IPF; this was a particularly common concern of patients using supplemental oxygen.

### Social participation

Most participants curtailed their social participation in engagements involving crowds of people for fear of "catching something [a respiratory illness that might lead to their demise]". Most patients went out to eat, to the theater, or to other social events much less frequently than before being diagnosed with IPF. Those in the later stages of the disease stayed in their homes almost exclusively. Many patients felt the need to try to hide the fact that they had a chronic illness when they were in public. The subjects were generally "more discriminating" with how they spent their time. This often translated into having difficulty "keeping up with certain relationships." Several patients felt like friends could not understand all that living with IPF entails. For nearly every patient (including those not yet needing supplemental oxygen), travel was considered extremely burdensome or, quite reluctantly, abandoned altogether.

### Mental and spiritual well-being

Several participants mentioned feeling sad, mainly in "anticipation of a decline in function". They commonly reported fear, worry, anxiety and panic and related these emotions to having IPF. IPF had the effect of "turning life upside-down" and causing them to "readjust life goals" and "refocus their lives". Many mentioned that IPF had become the "focal point" of their lives (and their spouses' and family members' lives as well). Some had difficulty with activities that required cognition or concentration. The effect of IPF on participants' spiritual well-being was generally positive; many of them became more contemplative, reflective, and had a stronger sense of their spiritual selves.

### Mortality

Not surprisingly, given the grave prognosis of IPF, the disease forced patients to "face reality", "recognize their mortality", and realize that " [they are] on the course of expiration". Participants yearned for more attention to end-of-life issues from the medical community. In general, they felt insecure about the dying process, afraid their symptoms would not be controlled toward the end of their lives and that the experience of death would be that of conscious suffocation. However, more than the dying process itself, many patients feared that they would be living a "worthless existence" toward the end of their lives. Many voiced concerns that they had several things that they wanted to do before dying. They wanted to get their "affairs in order" and most had "many preparations" to make in this regard.

### Comparison of the identified effects of IPF on quality of life with the content of existing instruments

Table 3 shows the numbers of items on each of the two non-IPF respiratory-specific measurement instruments (the CRQ-SAS and SGRQ) and the two generic instruments (the SF-36 and WHOQOL-100) which relate, in any way, to the domains that our participants identified as relevant to patients with IPF. The CRQ-SAS and SF-36 appear to have very limited coverage of the domains relevant to patients with IPF. Neither have items that focus on therapy, sleep, forethought, employment and finances, dependence, sexual relations, or mortality. Both of these instruments have items that address symptoms; however the scope of the CRQ-SAS is limited to assessing how breathless a respondent becomes while performing certain activities, and only one item on this instrument mentions cough. Certain items on the SF-36 ask about how physical health affects activities; however, there are no items that mention breathlessness or cough, which would make it impossible to differentiate their individual effects. Pain, which is also a domain on the SF-36, was not mentioned as part of our patients' disease experience. The wording of some items in the symptom domain of the SGRQ makes them irrelevant to patients with IPF; for example, they mention "wheezing", "attacks of chest trouble", or "chest condition" – effects and descriptions not identified by our participants. In addition, the SGRQ has no items that address sleep, dependence, family, sexual relations, or mortality. Patients in the current study identified multiple effects of IPF that were not covered at all on the WHOQOL-100, including several effects in our symptoms, therapy, forethought, dependence, and mortality domains.

**Table 3:** Comparison of the content/domain coverage of the CRQ-SAS, SF-36, SGRQ, and WHOQOL-100 with the domains identified in our conceptual framework

	**Number of Items**			
**Domains in conceptual framework**	**CRQ-SAS k = 20†**	**SF-36 k = 36†**	**SGRQ k = 50†**	**WHOQOL-100 k = 100†**
1. IPF Symptoms	5‡	14§	33 ∥	0
2. IPF Therapy	0	0	4**	0
3. Sleep	0	0	0	4
4. Exhaustion	4	4¶	2	4¶
5. Forethought	0	0	2	0
6. Employment and Finances	0	0	1	8
7. Dependence	0	0	0	0
8. Family	0	1	0	3
9. Sexual Relations	0	0	0	4
10. Social Participation	0	2	2	9
11. Mental and Spiritual Well-being	11	8	6	12
12. Mortality	0	0	0	0

**†**These columns show how each item from the CRQ-SAS, SF-36, SGRQ, and WHOQOL-100 maps onto the domains identified in this study; ‡One item mentions cough; §These items address the impact of physical health on activities but do not specifically address symptoms (e.g., breathlessness and cough); ∥Two items are about wheezing and two are about "attacks of chest trouble", making them irrelevant for patients with IPF; ¶These items ask about energy level and fatigue, but none of them pertain to exhaustion; **Items do not pertain specifically to IPF therapy; CRQ-SAS = Chronic Respiratory Questionnaire Self-Administered Standardized Format; SF-36 = Medical Outcomes Study Short-Form 36-item Instrument; SGRQ = St. George's Respiratory Questionnaire; WHOQOL-100 = World Health Organization 100-item Quality of Life Instrument.

## Discussion

In this study, we identified specific effects of IPF on patients' quality of life, by using patients' own perspectives. We grouped these specific effects into 12 conceptual categories, which compose both our conceptual framework of HRQL in IPF and might constitute provisional domains for a disease-specific measure. By eliciting patients' perspectives, we also have identified the reasons why existing generic and non-IPF respiratory disease-specific instruments are less than ideal for measuring QOL or HRQL in patients with IPF. An appropriate instrument must include items relevant to IPF patients and must tap important IPF-specific effects that are not captured well (or, in some cases, at all) by existing instruments.

According to patients, IPF significantly impairs quality of life. Symptoms of breathlessness and cough are extremely bothersome and limit physical activity, social participation, travel, and sexual relations. Fatigue, or more precisely, exhaustion is another effect of IPF that patients mention as occurring frequently and negatively impacting their lives. Interestingly, our patients were careful to make the distinction between breathlessness and low energy or exhaustion; they perceived the difference between the two quite clearly.

Most of our patients had to rearrange their lives quite extensively because of the effects of IPF. They had to take more time to prepare for the day, they used a lot of mental energy examining tasks to determine if they could complete them, and they were fearful of the impending need to depend on other people. Like many other patients with chronic or life-threatening illnesses, our patients took great comfort in realizing the love and support of their family members. While many patients recognized this positive aspect that living with IPF had on their relationships with their spouses and family members, several patients mentioned how the effects of this disease caused a great deal of tension between them and their loved ones. Not surprisingly, patients said they sometimes felt like a burden to other people, or they felt lazy because they were unable to do certain things (e.g., chores around the house).

Living with IPF also made patients reflect on their lives and their emotional selves. They were forced to think about things that they didn't necessarily want to think about (e.g., their own mortality and the effects that would have on loved ones left behind). Regarding death, patients wanted assurance that their symptoms would be controlled, that their passing would be peaceful, and that the dying process would occur on their own terms.

In the only study, other than the present one, that directly assessed IPF patients' perspectives, De Vries and colleagues [11] conducted three focus groups with a total of 10 IPF patients to assess the disease's impact on patient quality of life and to discuss the SGRQ and the WHOQOL-100. Their patients emphasized the physical limitations imposed by IPF and viewed the fatigue and social isolation caused by IPF as "serious problems". Other general areas perceived to be negatively affected by IPF included mobility, leisure activities, social relations, and working capacity – all areas included within the domains we have identified. Many of the basic findings of De Vries's and our study are consistent. However, perhaps because of the larger number of patients in the present study, or perhaps because of the somewhat more systematic and detailed analyses that we used, we identified additional effects of IPF that were not previously reported. Both De Vries's patients and (by inference) ours felt that the SGRQ did not adequately capture their disease experience, and the reasons are apparent when one inventories the SGRQ items in comparison with the effects we identified – as we have presented in Table 3.

In their study, De Vries and colleagues concluded that the WHOQOL-100 was well-suited to measuring QOL in patients with IPF, and that development of a disease-specific instrument for IPF was unnecessary [11]. We would agree that the WHOQOL-100 provides a useful measurement tool for many purposes, particularly if one is interested in comparisons across healthy populations or in those with a variety of health problems, rather than comparisons within a specific disease population. However, if the purpose is to measure changes in quality of life that may be associated with different stages of IPF, or that may be associated with different treatments of IPF, we would argue that because it is a generic instrument, the WHOQOL-100 is likely to have limited value. In fact, IPF patients in the study by De Vries and colleagues suggested that the WHOQOL-100 did not place sufficient emphasis on breathlessness, depression and social relationships. In addition, while the investigators stated that the WHOQOL-100 includes every general aspect of life that their patients mentioned, they did not detail the number of items that their patients found completely irrelevant nor how well patients believed that the purportedly relevant items captured their IPF-related circumstances.

To be useful and valid for a particular purpose in a given population, an instrument must not only tap relevant domains; even more importantly, the domains must be represented by relevant items that are in the correct range to adequately assess the population under study, and the instrument must possess the psychometric properties that substantiate its use in that population. There is no evidence to date that the WHOQOL-100 possesses the requisite sensitivity among IPF patients. That reason alone would render premature the conclusion that the WHOQOL-100 is adequate for studies in this population.

We would argue further that there is little reason for confidence that any of the existing measurement instruments that have been used in patients with IPF would be sufficiently sensitive for the purposes we are interested in – or that they would be more sensitive than an instrument specifically designed to address the concerns of patients with IPF. The instruments that we examined in this study focus on some aspects of disease that are not relevant to patients with IPF, they define or operationalize important domains in ways that make them less relevant for IPF, they miss potentially important domains altogether, their scales may be out of the range necessary to reflect the experiences of patients with IPF, and the steps between response options may be too great to capture important differences among IPF patients – or within the same IPF patient over time. All of these features tend to detract from the face validity of these instruments for IPF patients. If these instruments were used to evaluate IPF patients over time, their inclusion of items that cover less relevant topics may introduce variance into patients' scores that would tend to obscure, rather than reveal, changes in quality of life specifically associated with IPF or its therapy. Further, their omission of items on more relevant dimensions means that these instruments would not be able to reflect changes on those aspects at all.

While our study enrolled only 20 patients from one center, our sample included patients of varying age, with a broad range of disease duration, and representing the full spectrum of IPF disease. Some patients were diagnosed a short time before their focus group or interview, others were listed for lung transplantation at the time of the study, and some were in hospice care. While no study is absolutely free of bias, we attempted to minimize it by allowing the themes and items to emerge from the data.

## Conclusion

In this study, we conducted focus groups and in-depth interviews with a heterogeneous sample of IPF patients to identify specific effects of IPF on patients' lives. We used these patients' perspectives to develop a comprehensive conceptual framework for describing HRQL in this population. We identified 12 primary domains and numerous sub-categories and specific effects of IPF on the quality of patients' lives. We then examined how well four existing instruments covered these identified topics and found that there were several gaps and insufficiencies in these instruments' abilities to capture the effects of IPF on the quality of patients' lives. We suggest that a more appropriate instrument to measure HRQL in patients with IPF is needed and would be of great value.

## List of abbreviations

CRQ-SAS Chronic Respiratory Questionnaire Self-Administered Standardized Format

HRQL health-related quality of life

IPF idiopathic pulmonary fibrosis

QOL quality of life

SF-36 Medical Outcomes Study Short-Form 36-item Instrument

SGRQ St. George's Respiratory Questionnaire

WHOQOL-100 World Health Organization 100-item Quality of Life Instrument
